# HEMS inter-facility transfer: a case-mix analysis

**DOI:** 10.1186/s12873-018-0163-8

**Published:** 2018-05-16

**Authors:** Damien Di Rocco, Mathieu Pasquier, Eric Albrecht, Pierre-Nicolas Carron, Fabrice Dami

**Affiliations:** 1 Medical Student, Faculty of Medicine, University of Lausanne, Lausanne, Switzerland; 2 Emergency Department, Lausanne University Hospital (CHUV), Bugnon 46, 1011 Lausanne, Switzerland; 3 Department of Anaesthesia, Lausanne University Hospital, Lausanne, Switzerland

**Keywords:** Inter-facility transfer (IFT), Helicopter emergency medical services (HEMS), Case-mix, Over-triage

## Abstract

**Background:**

Helicopter emergency medical services (HEMS) are popular rescue systems despite inconsistent evidence in the scientific literature to support their use for primary interventions, as well as for inter-facility transfer (IFT). There is little research about IFT by HEMS, hence questions remain about the appropriateness of this method of transport. The aim of this study was to describe a case-mix of operational and medical characteristics for IFT activity of a sole HEMS base, and identify indicators of over-triage.

**Methods:**

This is a retrospective study on HEMS IFT over 36 months, from January 1st 2013 to December 31st 2015. Medical and operational data from the database of the Emergency Department of Lausanne University Hospital, which provides the emergency physicians for this helicopter base, were reviewed. It included distance and time of flight transport, type of care during flight, and estimated distance of transport if conducted by ground.

**Results:**

There were 2194 HEMS missions including 979 IFT (44.6%). Most transfers involved adults (> 17 years old; 799 patients, 81.6%). Forty patients (4.1%) were classified as having benefitted from resuscitation or life-saving measures performed in flight, 615 (62.8%) from emergency treatment and 324 (33.1%) from simple clinical examination. The median distance by air between hospitals was 35.4 km. The estimated median distance by road was 47.7 km. The median duration time from origin to destination by air was 12 min.

**Conclusions:**

This case-mix of IFTs by HEMS presents a high severity. There are many signs in favour of over-triage. We propose indicators to help choosing whether HEMS is the most appropriate mean of transport to perform the transfer regarding patient condition, geography, and medical competences available aboard ground ambulances; this may reduce over-triage.

## Background

Helicopter emergency medical services (HEMS) are popular rescue systems despite inconsistent evidence in the scientific literature to support their use for primary interventions, as well as for inter-facility transfer (IFT) [1–5]. For IFT, the main added values of the helicopter are speed of patient transport, and sometimes medical competences if not available in ground ambulances (GA). IFT can either upgrade the level of care (to a trauma centre or university hospital) or downgrade it (to make room in those trauma centres or university hospitals) [6].

In contrast to direct transport from the scene of injury, there has been much less research about IFT by HEMS; hence, questions remain about the appropriateness of this method of transport. Furthermore, research on this topic has been mainly limited to specific types of disease (STEMI, stroke, spinal injury) and not on whole HEMS case-mixes [7, 8]. To the best of our knowledge, there is no generally accepted guideline that would help choose the most appropriate transport method for IFT, nor definition of over-triage when using the helicopter to perform those IFTs.

The aim of this study is to describe a single HEMS IFT case-mix and its severity, and identify over-triage through operational and medical indicators.

## Methods

### Setting.

The State of Vaud (western Switzerland) has one trauma centre (Lausanne University Hospital), seven regional hospitals and many private clinics that are distributed equally over its territory. Most hospitals have a GA which they can use for transfers. All GAs are staffed with at least one paramedic. They use State protocols for autonomous intravenous access, cardiopulmonary resuscitation procedures, defibrillation and emergency medication administration. They are not allowed to manage upper airway disposals (intubation, laryngeal mask or tube) or continuous drug infusions (vasopressors, anaesthesia and sedation); these procedures require the presence of an emergency physician (EP). EPs are scarcely available to conduct IFT by GA as hospitals want to keep these scare resources within their emergency department. Therefore, HEMS which are staffed with their own EP and paramedic are regularly used to perform those transfers. Switzerland is very well covered for its area of 41,300 km^2^ (16,000 mile^2^), with 20 medically equipped helicopters from private companies during the daytime, and 8 during the night. In this State, requests for IFT by the helicopter are made by the hospital medical team in charge of the patient; there is no triage either from the HEMS companies or from dispatch centres regarding secondary transport.

### Study design.

This was a retrospective study, carried out on data from January 1st 2013 to December 31st 2015 (36 months). All data were extracted from the database of the Emergency Department of the Lausanne University Hospital which staffs the EPs for this HEMS. Pre-hospital medical charts are completed by EPs and then checked by medical supervisors.

### Measurements and outcomes.

Demographics (age and sex), diagnosis and operational data (distances, origin and destination, date, time of day and duration of flight) were collected. Patients who were 17 years of age and under were included in a paediatric subgroup. Outcome at 48 h (mortality, ICU or ward stay, discharge) was obtained from hospital charts. Diagnosis was grouped into nine clinical categories (*heart and vessel disease*, *traumatology*, *neurology*, *pneumology*, *obstetrics*, *paediatrics*, *toxicology*, *psychiatry* and *miscellaneous*). Patient care provided during transfer was classified into three care categories by EPs: *simple clinical examination*, *emergency treatment* and *resuscitation or life-saving manoeuvres*. Simple clinical examination consisted of simple monitoring of the patient during the flight. Emergency treatment consisted of administering any medication (continuously or not) including fluid resuscitation, even if it was started in the hospital, or to pursue ongoing ventilation. Resuscitation consisted of gestures and manoeuvres necessary to maintain the patient’s life, such as cardiac massage, defibrillation or intubation. Specific treatments requiring the presence of an EP in this system (continuous drug infusion or airway management) were collected. Distances of journeys by road were calculated using Google Maps®; major differences between flight and ground distances for the same IFT may reveal geographical constraints (e.g. mountains, water).

Data were integrated into an Excel® spreadsheet, and processed and analysed using Stata© (Stata, Statistical Software 14.2, Stata Corporation, College Station, TX, USA).

## Results

**Fig. 1:**
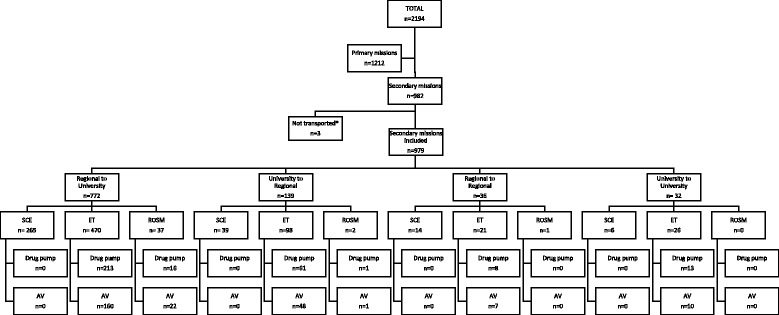
Inter-facility transfer flowchart. SCE: simple clinical examination, ROSM: resuscitation or life-saving manoeuvres, ET: emergency treatment, AV: assisted ventilation (intubated/tracheotomised). *not transported: died before take-off

During the study period, there were 2194 HEMS missions including 982 IFT (44.8%). Three patients were excluded as they died before take-off; the final group included 979 patients (Fig. 1) (Table 1). Most missions involved adults (> 17 years old) (799; 81.6%). There were 772 (78.8%) transfers from regional hospitals to a university hospital, 139 (14.2%) from a university hospital to a regional hospital, 36 (3.7%) from a regional hospital to another regional hospital and 32 (3.3%) from a university hospital to another university hospital. Trauma patients represent 15.5% of the case-mix. Forty patients (4.1% of total) were classified as having benefitted from *resuscitation or life-saving measures* performed in flight, 615 (62.8%) from an *emergency treatment* and 324 (33.1%) from a *simple clinical examination*. Table 2 lists the fate of all patients at 48 h, regarding the type of care performed *en route*.

**Table 1:** Patients’ clinical characteristics including demographics, medical equipment according to the type of care provided, and clinical category (*n* = 979)

Demographic	
All	
n (%)	979 (100)
Gender male, n (%)	594 (60.7)
Age (mean ± SD) (range)	50.7 ± 26.9 (1–95)
Adults	
n (%)	799 (81.6)
Gender male, n (%)	487 (49.7)
Age (mean ± SD) (range)	60.8 ± 18.1 (18–95)
Paediatric patients	
n (%)	180 (18.4)
Gender male, n (%)	107 (10.9)
Age (mean ± SD) (range)	6.1 ± 5.1 (1–17)
Care category (= n) (%)	
Simple clinical examination	324 (33.1)
*Intubated before transfer*	0 (0)
*Tracheotomized before transfer*	0 (0)
*Infusion pumps*	0 (0)
Emergency treatment	615 (62.8)
*Intubated before transfer*	205 (20.9)
*Tracheotomized before transfer*	20 (2.0)
*Infusion pumps*	295 (30.1)
Resuscitation or life-saving measures	40 (4.1)
*Intubated before transfer*	23 (2.3)
*Tracheotomized before transfer*	0 (0)
*Infusion pumps*	17 (1.7)
Clinical category (= n) (%)	
Heart/vessel disease	252 (25.7)
*Myocardial infarct*	103 (10.5)
*Chest pain*	38 (3.9)
*Cardiac arrest*	28 (2.9)
*Cardiac insufficiency*	17 (1.7)
*Aortic abdominal aneurysm ruptured*	14 (1.4)
*Other*	52 (5.3)
Traumatology	152 (15.5)
*Mild TBI (GCS 14–15)*	29 (3)
*Blunt abdominal trauma*	24 (2.5)
*Moderate TBI (GCS 9–13)*	13 (1.3)
*Severe TBI (GCS 3–8)*	13 (1.3)
*Other trauma*	73 (7.5%)
Neurology	123 (12.6)
*Stroke*	85 (8.7)
*Status epilepticus*	16 (1.6)
*Epilepsy*	14 (1.4)
*Other*	8 (0.8)
Pneumology	85 (8.7)
Obstetrics	58 (5.9)
Paediatrics	46 (4.7)
Toxicology	25 (2.6)
Psychiatry	3 (0.3)
Miscellaneous	235 (24)

*SD* standard deviation, *GCS* Glasgow Coma Scale, *TBI* traumatic brain injury

*Continuous drugs used: norepinephrine, dopamine, dobutamine, propofol, clonidine, sodium nitroprusside, labetalol, alteplase, heparin, isosorbide dinitrate, nitroglycerin, atosiban

**Table 2:** Patients’ 48 h outcome according to the type of care provided

		Type of care		
Outcome at 48 h (= n)	Total (n = 979)	Simple clinical examination (*n* = 324) (33.1%)	Emergency treatment (*n* = 615) (62.8%)	Resuscitation or life-saving measures (*n* = 40) (4.1%)
Hospitalized in intensive care unit	425 (43.4)	75 (7.7)	325 (33.2)	25 (2.6)
Hospitalized in intermediate care unit	196 (20)	96 (9.8)	96 (9.8)	4 (0.4)
Hospitalized in ward division	181 (18.5)	70 (7.2)	108 (11.0)	3 (0.3)
Transfer to another institution	88 (9)	42 (4.3)	45 (4.6)	1 (0.1)
Hospitalized and returned home	44 (4.5)	34 (3.5)	10 (1.0)	–
Not hospitalized, discharged	9 (0.9)	4 (0.4)	5 (0.5)	–
Died: 0–1 h (after admission)	3 (0.3)	1 (0.1)	–	2 (0.2)
Died: 1–6 h	3 (0.3)	–	3 (0.3)	–
Died: 6–24 h	19 (1.9)	–	14 (1.4)	5 (0.5)
Died: 24–48 h	11 (1.1)	2 (0.2)	9 (0.9)	–

### **Operational characteristics** (Table 3).

The median distance by air between hospitals was 35.4 km. The median distance by road calculated using Google Maps® was 47.7 km. The overall duration from origin to destination by air was 12 min.

**Table 3:** Operational characteristics

	HEMS	Road (estimated)
Total missions, n	979	979
Median distance (km) (IQR)	35.4 (22.5-–40.2)	47.7 (30.5–71.1)
< 30 km, n (%)^a^	342 (34.9)	241 (24.6)
≥ 100 km, n (%)^¶^	37 (3.8)	70 (7.1)
Median duration (min) (IQR)	12 (10–15)	
Flow of transfers & clinical classification		
University to university	32 (3.3%)	
*Simple clinical examination*	6 (0.6)	
*Emergency treatment*	26 (2.7)	
*Resuscitation or life-saving measures*	0 (0)	
Regional to regional	36 (3.7%)	
*Simple clinical examination*	14 (1.4)	
*Emergency treatment*	21 (2.1)	
*Resuscitation or life-saving measures*	1 (0.1)	
University to regional	139 (14.2%)	
*Simple clinical examination*	39 (4.0)	
*Emergency treatment*	98 (10.0)	
*Resuscitation or life-saving measures*	2 (0.2)	
Regional to university	772 (78.8%)	
*Simple clinical examination*	265 (27.1)	
*Emergency treatment*	470 (48.0)	
*Resuscitation or life-saving measures*	37 (3.8)	
Night mission 7 pm–7 am	361 (36.9%)	

IQR: interquartile range

^a^Expert recommendation by Kim et al. study, ^¶^expert recommendation by Kristiansen et al. study

There were 270 (27.6%) transfers during weekends, and 361 (36.9%) night transfers (7 pm–7 am).

## Discussion

The decision to use HEMS is a sensitive topic, and there is no unambiguous evidence in favour or against its use for IFT in the literature; studies are carried out in different health policy settings and in different geographical environments; their results cannot be directly transposed to other EMS. Some studies have described factors (e.g. distance, geography) that influence the use of HEMS for IFT [9–11]; some demonstrated either a survival advantage [1, 12, 13] or disadvantage [2, 11] for injured patients. Moreover, most of these studies take into account only one category of pathology and not the whole case-mix, as this study proposes [8, 9].

The analysis of the case-mix, the first aim of the study, shows that 425 (43.4%) patients were hospitalised in an ICU at 48 h, which demonstrates the severity of this case-mix. There were few trauma cases in this case-mix, which could be explained by efficient sorting performed during on-scene missions, and a consequently low retransfer rate, as previously published in this HEMS [14]. Out of the 974 IFT, 772 (79%) patients were transported from a regional hospital to a university hospital (upgrading the level of care).

The measure of over-triage, the second aim of the study, is complex and must take into account many different indicators. For example, upgrading the level of care or the severity conditions at 48 h mentioned above are not indicators that can justify the use of HEMS by themselves.

If speed is needed by the referral team, then distance by air and ground have to be taken into account. If they are very different (e.g. affected by mountains, water), the probability that HEMS may be faster is high. Regarding this issue, some have proposed using distances between hospitals or the expected duration of transfer to justify the use of HEMS [15, 16], but there is no consensus on a cut-off distance or time for which the helicopter should be used [5]. Kristiansen et al. showed an increase in the use of HEMS proportional to the distance to be covered, and a decrease in mortality for transfers by HEMS ≥100 km [15]. Another study recommends considering HEMS for distances ≥30 km by road for IFT [16]. If we transpose these values to our study, we note that 738 (75%) missions were above the 30 km proposed limit, and 70 (7%) above the 100 km limit. It should however be highlighted that although a helicopter can cover a greater distance than a GA in a given time, it is not always the fastest method of transfer [5]. Indeed, it takes longer to install a patient in a helicopter than in a GA. Take-off and landing procedures also take longer than starting an ambulance engine [17]. In this area, although hospital landing zones are open 24/24, they are often located on roof tops or at some distance from emergency departments. Finally, it should be mentioned that most hospitals have a GA service nearby, which may be at the patient’s bedside somewhat quicker than HEMS. When looking for speed, the estimated global time of transfer from the call to the arrival at destination should be estimated by air versus ground.

In some EMS, GA medical competences are sometimes estimated to be insufficient to handle the patient and therefore HEMS is requested. This is the case in this study, as paramedics cannot handle intubation, mechanical ventilation or continuous drugs. When looking at the *simple clinical examination* group, the main possible added value of HEMS was speed of transport to exclude and/or treat a time-sensitive condition (i.e. STEMI, stroke, angiography, or neurosurgery) after having received all necessary treatment in the local hospital. When analysing in detail the diagnosis declared by EPs for this category, we can retrospectively hypothesise that some of them may not have needed HEMS as advanced medical competences were not required (head trauma with GCS 14, spine trauma without neurological deficit, pneumonia, intoxication, alcohol abuse). When looking ate the *emergency treatment* group, the vast majority did not benefit from ventilation or continuous drug treatment. The medical files were not retrospectively checked to determine whether continuous treatment could have been interrupted for the duration of transfer, allowing paramedics to take care of the patient from this category without EP.

HEMS should be used for IFT if medical competences that exceed GA competences are needed or may be needed during transfer, or if speed is needed and the estimated time from call to arrival at the destination is faster with a helicopter. In the setting described, the hospital physician in charge of the patient performs this triage, but they are often not aware of paramedic competences and do not have the information on GA availability. Ideally, a dispatch centre should decide whether to allow HEMS transfers or not based on the need of HEMS for primary missions, the patient’s condition, the suspected pathology and time gained by using HEMS for IFT. This would require advanced medical competences within the dispatch centre; it may also allow the treatment to be simplified wherever possible, to enable the GA to take care of the patient.

In the future, to better understand the use of HEMS for IFT and thus be able to propose new guidelines, it should be mandatory to prospectively document the reason why physicians choose HEMS instead of GA, in particular if it is a matter of speed or level of care. It should also be mandatory to evaluate whether ongoing continuous treatment can be stopped briefly or not. The dispatch centre should be capable of deciding whether to use HEMS for IFT rather than in-hospital physicians; all of those measures may contribute to a reduction in the over-use of HEMS for IFT.

The results point out that there are numerous signs of over-triage in this case-mix, regarding either the flight distances, clinical categories, medical procedures performed during flight, and diagnosis of patients. However, we lack two critical pieces of information to fully circumscribe the issue: the reason why HEMS is requested (speed? Medical competences?) and the estimate time saved using HEMS versus GA for each IFT, taking into account the time from the alarm and the fact that an ambulance may already be at the hospital.

There will always be a certain amount of over-triage in the use of HEMS for transfer and we should accept it. The question, as always, is how much is too much?

### Limitations.

This is a monocentric and retrospective study. The data available could not define whether primary missions needing HEMS had to go by ground because of transfer activity with the helicopter. The reason why physicians required HEMS instead of a GA for transfer is not known, as it is not documented in hospital or pre-hospital charts. The category used to describe the treatment received during flight is not a validated standard. Air and ground distances were estimated using Google Maps®. The elapsed time between HEMS activation and arrival at the patient’s bedside was not described, as some transfers were ‘scheduled’, meaning that the crew was asked to take off within a specific delay (30–90 min). Only the duration of transport by HEMS was measured, rather than the entire process from the alarm to the arrival of HEMS at the receiving facility. No direct comparison was made with a GA; therefore, we cannot assess whether using HEMS for those transfers was quicker than if they had been performed by GA. This study did not intend to measure under-triage, as this would have required the operational characteristics of ground IFTs.

This study took place in a specific setting (geography, paramedics’ autonomy, absence of EPs in GAs), and may not be applicable elsewhere.

## Conclusions

The case-mix of IFTs by HEMS analysed presents a high severity. There are many signs in favour of over-triage. We propose indicators to help to determine whether HEMS is the most appropriate to perform the transfer regarding patient condition, geography, and medical competences available on-board GA; this may reduce over-triage.

## Abbreviations

EMS: Emergency medical service
EP: emergency physician
GA: Ground ambulance
HEMS: Helicopter emergency medical service
ICU: Intensive care unit
IFT: Inter-facility transfer
ISS: Injury severity score
STEMI: ST-elevation myocardial infarction
